# Tunnelling anisotropic magnetoresistance due to antiferromagnetic CoO tunnel barriers

**DOI:** 10.1038/srep15498

**Published:** 2015-10-21

**Authors:** K. Wang, J. G. M. Sanderink, T. Bolhuis, W. G. van der Wiel, M. P. de Jong

**Affiliations:** 1 NanoElectronics Group, MESA+ Institute for Nanotechnology, University of Twente, P.O. Box 217, Enschede, 7500AE, The Netherlands

## Abstract

A new approach in spintronics is based on spin-polarized charge transport phenomena governed by antiferromagnetic (AFM) materials. Recent studies have demonstrated the feasibility of this approach for AFM metals and semiconductors. We report tunneling anisotropic magnetoresistance (TAMR) due to the rotation of antiferromagnetic moments of an insulating CoO layer, incorporated into a tunnel junction consisting of sapphire(substrate)/fcc-Co/CoO/AlO_x_/Al. The ferromagnetic Co layer is exchange coupled to the AFM CoO layer and drives rotation of the AFM moments in an external magnetic field. The results may help pave the way towards the development of spintronic devices based on AFM insulators.

In spintronics, the spins of electrons and holes are used in addition to their charge to process and store information, offering new prospects for making electronic devices. Ferromagnetic (FM) materials are often used to generate spin-polarized charge currents or pure spin currents^1^,^2^,^3^. Antiferromagnetic (AFM) materials are also ubiquitous in spintronic devices, but are used mostly in a passive role, namely to induce exchange bias in an adjacent ferromagnetic (FM) layer^4^,^5^. Recent studies have shown that AFM materials can also actively *determine spin-dependent transport phenomena* in spintronic devices, which broadens the application horizon for these materials considerably and offers additional advantages, since AFM materials hardly produce any stray magnetic fields, and are much more abundant than ferromagnets. So far, however, active manipulation of spin transport has been achieved only with metallic or semiconducting AFM materials. Here, we focus on a new tunnelling anisotropic magnetoresistance (TAMR) effect, which is produced by an insulating AFM CoO layer. Coupling between this AFM insulator and an adjacent FM Co electrode produces an exchange spring effect that results in the rotation of both FM and AFM moments under application of an external magnetic field, yielding a robust TAMR effect up to 2.5%. Our results demonstrate that AFM insulating tunnel barriers may be used to generate magnetoresistance in tunnel junctions, and help pave the way for the development of novel AFM-based spintronic devices.

TAMR is a phenomenon that relies on the anisotropy of the spin-orbit coupling (SOC) in magnetic crystals^6^. Due to this anisotropy, the tunnelling DOS in an MTJ is modulated by rotating the spin quantization axis, which results in different resistance values for different magnetization directions. The effect can be observed in tunnel junctions with a single FM contact and a non-magnetic counter electrode separated by an insulating or semiconducting tunnel barrier^7^,^8^. It has been proposed recently that such effects should be equally well present in AFMs, and experimental confirmation has been realized in tunnel junctions consisting of a bi-layer FM/AFM-metal electrode (NiFe/IrMn), a tunnel barrier, and a non-magnetic counter-electrode^9^,^10^,^11^. Very recently, anisotropic magnetoresistance (AMR) in an AFM *semiconductor* Sr_2_IrO_4_ has been reported, using current-perpendicular-to-plane measurements of heterostructures comprising AFM Sr_2_IrO_4_ layers and FM La_2/3_Sr_1/3_MnO_3_ electrodes^12^. Here, we report TAMR effects of up to 2.5% in tunnel junctions that contain *insulating* AFM CoO tunnel barriers, which are exchange coupled to FM Co electrodes (see Fig. 1). We will show that the TAMR effect is governed by the rotation of the AFM moments at the Co/CoO interface rather than simply the FM moments of the Co electrode. Ever since the discovery of the exchange bias effect in Co/CoO core-shell nanoparticles by Meiklejohn and Bean in 1956^13^,^14^, the impact of the Co/CoO interfacial spin system on magnetization reversal processes has been intensively studied, in particular using anisotropic magnetoresistance (AMR) measurements of bi-layer Co/CoO structures^15^,^16^. Our results show that these interfacial spin systems also hold great promise for the active manipulation of spin polarized transport.

## Results

### Anisotropic magnetoresistance of Co/CoO bilayers

**Figure 1:**
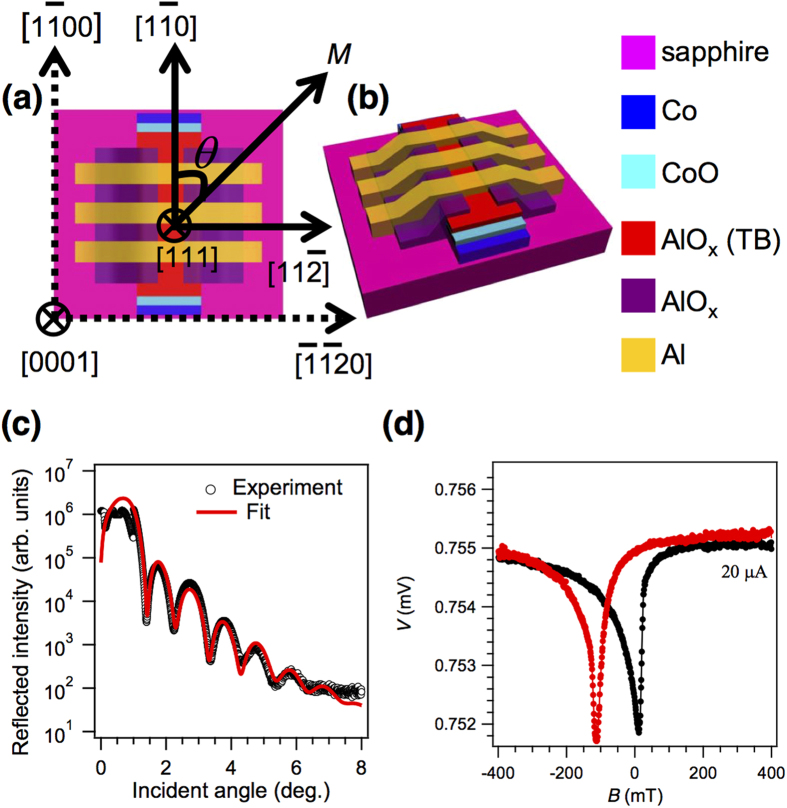
Schematic device structure and characterization of the Co/CoO bilayer. (**a**) Top view of the spintronic device with structure sapphire/Co(4.5 nm)/CoO(4 nm)/AlO_x_(3.3 nm)/Al(35 nm), with crystallographic directions shown of the single crystalline sapphire substrate and the epitaxial 4.5-nm Co thin film. (**b**) Three-dimensional view of the device structure. (**c**) XRD reflectivity measurement. (**d**) Anisotropic magnetoresistance of the Co/CoO bilayer (resistance ~37.5 Ω) measured at 5 K under a *dc* bias *I *= 20 μA after field cooling in a 800 mT magnetic field. The black (red) trace corresponds to a magnetic field sweep from negative (positive) to positive (negative) values.

Figure 1(d) displays a typical current-in-plane (CIP) AMR measurement of the Co electrode of a tunnel junction. The current and the sweeping magnetic field were both in parallel with the easy axis of the Co electrode. The magnetic reversal process can be clearly observed, and the magnetic hysteresis loop exhibits a considerable shift away from zero field: The coercive fields are approximately equal to 12 and −112 mT, respectively, for the two opposite magnetic field sweeping directions. This shows that exchange coupling is present at the Co/CoO interface. In addition to a rigid shift of the hysteresis loop, which results from the unidirectional exchange bias field, a clear magnetization reversal asymmetry is present, typical for exchange-coupled Co/CoO bilayers and other FM/AFM systems. It has been shown previously that the mechanism for magnetization reversal in Co/CoO stacks is different for field sweep directions that are parallel or antiparallel with the exchange bias field. If the field is increased in the direction of the exchange bias field, switching occurs via rotation of moments, whereas in the opposite direction domain wall nucleation and propagation occurs^16^. Domain state model calculations have been reported, suggesting that the relative orientation of the magnetic field and the AFM magnetic easy axis (which may not coincide with the FM easy axis) may play an important role in the magnetization reversal process^17^.

### Tunneling anisotropic magnetoresistance measurements using sweeping magnetic fields

**Figure 2:**
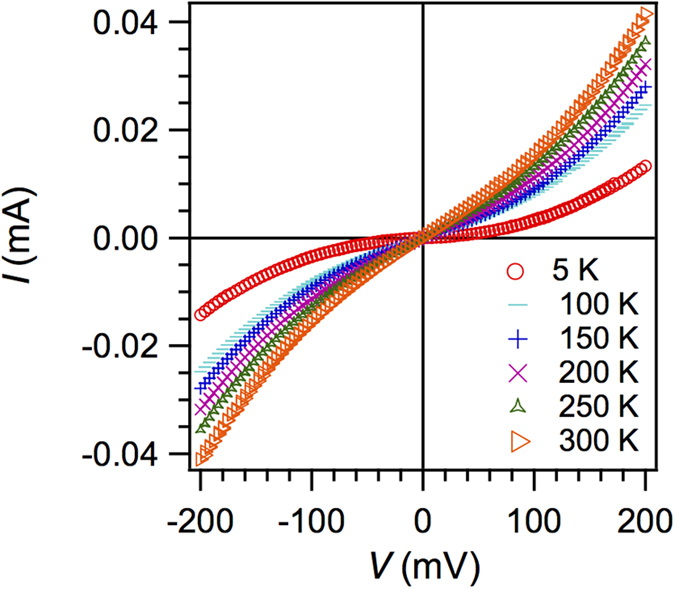
Temperature dependent *IV*–curves. The tunnel junction, consisting of sapphire/Co(4.5 nm)/CoO(4 nm)/AlO_x_(3.3 nm)/Al(35 nm), features a static junction resistance of 4.8 kΩ at 5 K and a bias voltage of 200 mV.

We now address magnetotransport data measured in the current-perpendicular-to-plane (CPP) geometry through the Co/CoO-based tunnel junction of the same device, starting with the analysis of a basic electron transport measurement. Figure 2 shows the temperature dependence of the *I*–*V* characteristics. All the plots depict nonlinear and quasi-symmetric *I*–*V* characteristics, while the variations of junction resistance with the temperature are modest. This indicates that electron tunnelling is dominant. Here, and also for the following measurements, positive and negative bias voltage (or current) refer to electrons tunnelling from Co to the Al counter-electrode and *vice versa*.

**Figure 3:**
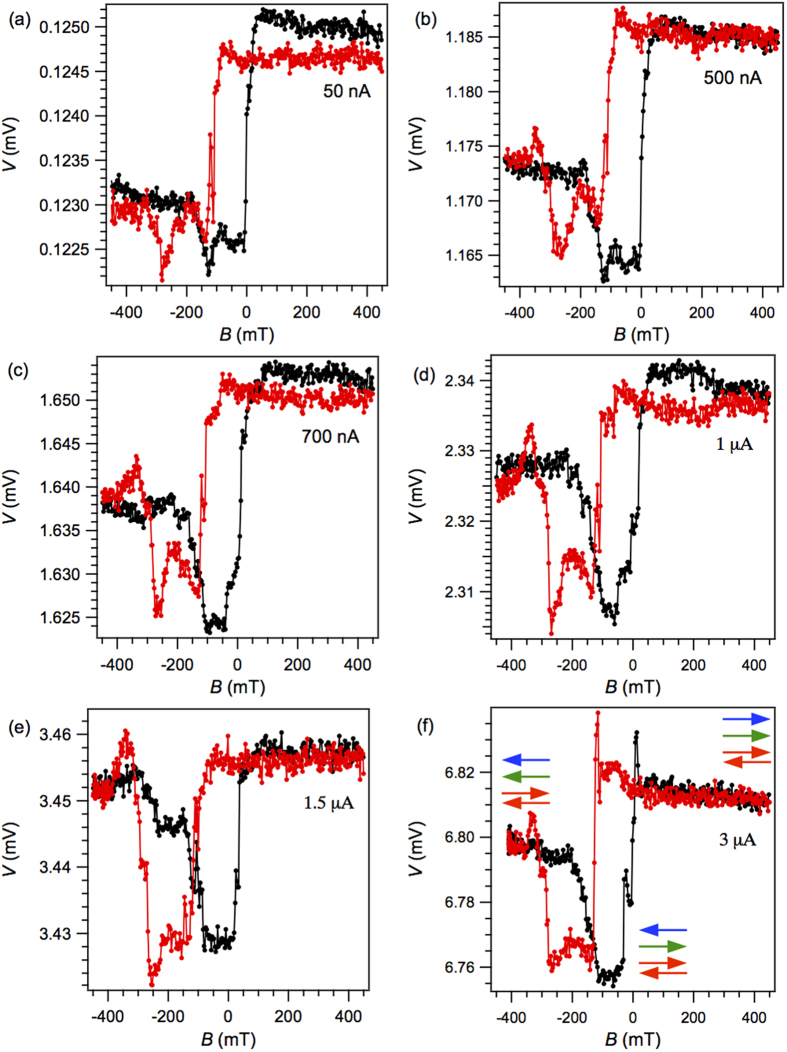
Positive bias-dependence of the in-plane TAMR. (**a**–**f)** Magnetic field sweeps were performed at 5 K under application of a constant bias current of (**a**) 50 nA, (**b**) 500 nA, (**c**) 700 nA, (**d**) 1 μA, (**e**) 1.5 μA and (**f**) 3 μA, where the orientation of Co ferromagnetic moments, interfacial moments, and bulk CoO antiferromagnetic moments for specific resistance states is indicated with blue, green and red arrows, respectively. The black (red) trace corresponds to a magnetic field sweep from negative (positive) to positive (negative) values.

Figure 3(a)–(f) show TAMR measured at 5 K upon sweeping the magnetic field *B*. It is clear that the magnetoresistance (which amounts to about 2% at *I* = 50 nA) observed in CPP measurements through the tunnel junction is dramatically different from the AMR signal measured in the CIP configuration. This indicates that the TAMR measurements are governed by the rotation of AFM moments at the Co/CoO interface, rather than the FM moments of the Co electrode alone. The clearest indication that AFM moments are involved is the observation of different resistance states at large negative *versus* large positive magnetic field values. This behaviour is qualitatively similar to that of tunnel junctions comprising FM/AFM metal electrodes^12^, and has been attributed to incomplete rotation (i.e. smaller than 180°) of the AFM moments^9^. Clearly, the insulating AFM CoO barrier produces a similar effect in our junctions. The ferromagnetic layer reverses its magnetization upon sweeping the magnetic field, leading to a rotation of the interfacial AFM moments due to the exchange coupling at the interface, which is however counteracted by the coupling to the “bulk” AFM ordered spin system. Consequently, the rotation of the AFM moments is incomplete, consistent with the observation of different resistance values at large magnetic fields of opposite sign. However, bias dependent TAMR measurements reveal additional complexity in the magnetization reversal of the interfacial spin system, as shown below.

Figure 3(a), corresponding to a bias current of 50 nA, displays a quasi-square magnetic hysteresis loop. The sharp switching events correspond to the coercive field values of the Co layer (see Fig. 1(d)). A clear shift of the centre of the hysteresis loops away from *B* = 0 can be observed, consistent with the AMR measurement, which is due to the exchange bias effect. When the bias current is increased from 50 nA to 3 μA, as shown in Fig. 3(a)–(f), the quasi-square magnetic hysteresis loop gradually turns into a spin-valve like signal with plateaus at intermediate magnetic field values. These plateaus may be associated with a metastable orientation of a subsystem of AFM moments at the Co/CoO interface.

It is known that interfacial moments in exchange coupled FM/AFM layers can be subdivided in different classes, exhibiting different magnetization behavior. Typically only a small fraction of interfacial moments is rigidly pinned to the bulk AFM lattice, while others rotate with the FM magnetization^15^,^18^. It might thus be inferred that the spin-valve like plateaus are related to a fraction of interfacial moments that undergo magnetization reversal upon sweeping the magnetic field like the ferromagentic moments, but at different magnetic field strengths, as indicated in Fig. 3(f).

Since the corresponding TAMR features become more pronounced as the bias window is increased, the electronic states associated with these particular moments must reside below *E*_F_. It is interesting to note that these features appear in the magnetic field sweep regions where the FM moments are antiparallel to the exchange bias field. At *I* = 1.5 μA (Fig. 3(e)), the magnetoresistance is approximately equal to be 0.8% using the definition MR(%) = (R_HVS_ − R_LVS_)/R_LVS_ × 100, where the subscripts indicate high and low voltage/resistance states (HVS and LVS).

**Figure 4:**
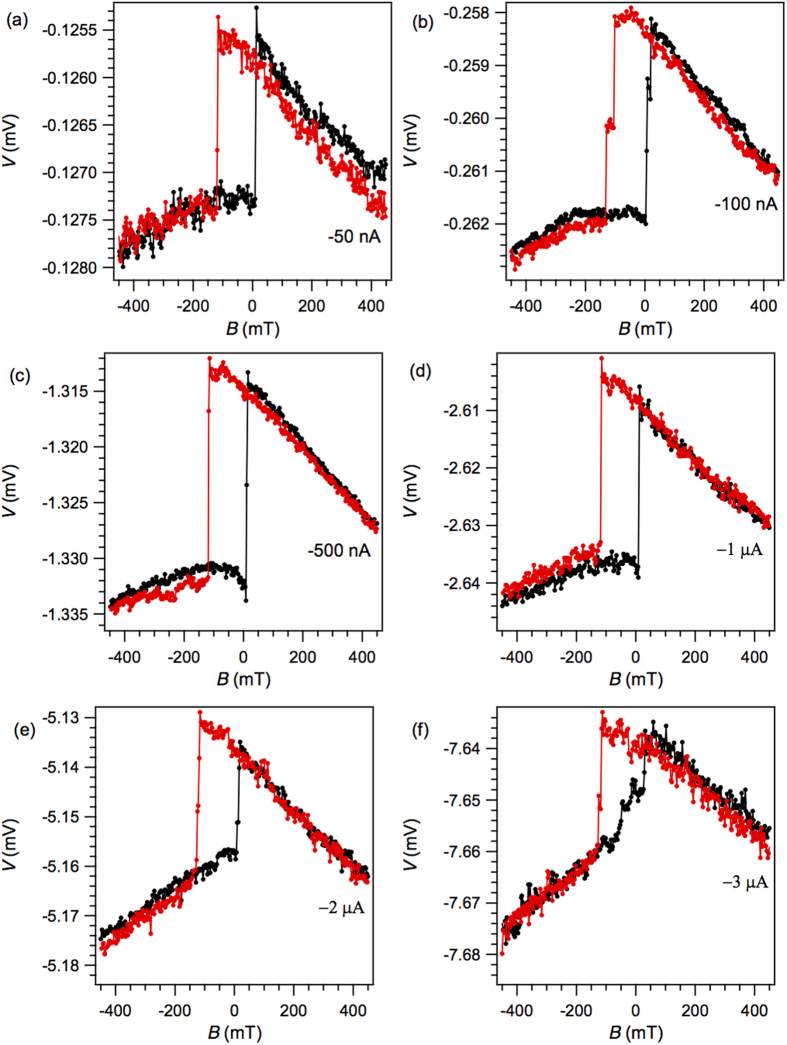
Negative bias-dependence of the in-plane TAMR. (**a**–**f)** Magnetic field sweeps were performed at 5 K under application of a constant bias current of (**a**) −50 nA, (**b**) −100 nA, (**c**) −500 nA, (**d**) −1 μA, (**e**) −2 μA and (**f**) −3 μA. The black (red) trace corresponds to a magnetic field sweep from negative (positive) to positive (negative) values.

The TAMR changes significantly when the polarity of the bias current is reversed, such that electrons tunnel from Al to Co. Figure 4(a)–(f) depict the corresponding magnetoresistance measurements for the same device. In these plots, the quasi-square magnetic hysteresis loops can still be observed, with sharp switching events corresponding to the coercive field values of the Co electrode, and different resistance values at high negative *versus* positive magnetic fields. However, no additional features are present.

### Tunneling anisotropic magnetoresistance measurements using static magnetic fields

**Figure 5:**
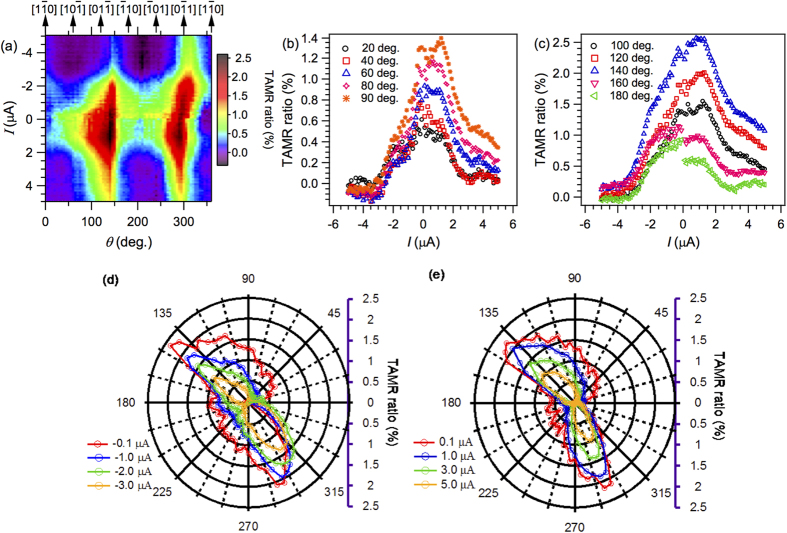
TAMR measured at 5 K under application of a constant magnetic field of 800 mT. (**a**) Contour plot of the TAMR ratio as a function of applied bias current and in-plane magnetization angle. The color in the contour plot represents the magnitude of the TAMR ratio in percent (see color bar). (**b**,**c**) TAMR versus bias current for several different angles from 0° to 180°. (**d**,**e**) Angle dependence of the TAMR for negative and positive bias current, respectively.

We now address TAMR measurements for the same device obtained by rotating a 800 mT magnetic field along different in-plane angles, see Fig. 5(a)–(e). The 2-D image plot of Fig. 5(a) displays TAMR ratios as a function of bias current (within the same bias window as previously) and in-plane angle *θ*. Since the fcc-Co thin film grows epitaxially on the monocrystalline sapphire substrate, the in-plane crystallographic axis of the Co layer can, thus, be indexed as shown at the top axis. From our previous work on reference structures consisting of sapphire/Co(8 nm)/AlO_x_(3.3 nm)/Al(35 nm), we have found that the epitaxial fcc-Co layer exhibits in-plane uniaxial magnetocrystalline anisotropy, with the easy and hard axes pointing along two perpendicular crystallographic axes 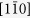 and [11-2] (Fig. 1(a))^1^. The largest in-plane TAMR ratio in such reference structures was found to be 7.5%. In the present case, with the inclusion of an AFM CoO layer, the TAMR ratio is suppressed and is lower than 2.5%. The TAMR ratios are all positive, which means that the reference direction 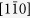, corresponding to the easy axis, exhibits the smallest voltage/resistance. Figure 5(b,c) show plots of the TAMR bias dependence at selected angles. Both plots display non-symmetric curves with respect to zero bias current. For negative bias currents, corresponding to electrons flowing from Al to Co, the TAMR decreases monotonically with increasing current, for all angles. For positive bias currents, the maximum TAMR is always at finite bias current close to 1.5 μA, corresponding to a bias voltage of about 3.4 mV. Comparing with Fig. 3, this coincides with the bias current for which large TAMR effects due to metastable orientation of AFM moments at the Co/CoO interface were observed during magnetic field sweeps. These observations may be ascribed to a resonant tunnelling process for this bias window, within which some interfacial states are sampled corresponding to uncompensated AFM spins. Figure 5(d,e) show the TAMR angle dependence at negative and positive bias currents, respectively. In contrast to Co/AlO_x_/Al reference devices^1^, the TAMR signal does not show two-fold rotational symmetry, consistent with the notion that the effect originates from the AFM CoO layer.

## Discussion

The uncompensated AFM spins at the Co/CoO interface thus not only play an important role in the magnetization reversal mechanism, reflected in e.g. coercivity changes and exchange bias^15^, but also in the tunnelling processes. Using X-ray magnetic circular dichroism (XMCD), X-ray magnetic linear dichroism (XMLD) and X-ray photoemission electron spectroscopy (XPEEM), magnetic domain structures have been imaged and FM/AFM interactions were studied for several representative heterointerfaces, like Co/NiO and Fe/NiO, as well as Co/CoO^15^,^18^,^19^,^20^. An important property of such structures is that AFM magnetic domain walls are wound up along the vertical growth directions of the heterostructures as the FM magnetization is rotated, which is commonly referred to as the exchange spring effect. In the (angle-dependent) TAMR measurements, the rotation of both FM and AFM moments modulate the Co/CoO interfacial tunnelling DOS via the anisotropy of the SOC of the corresponding electronic states. In the measurements of Fig. 5, the FM fcc-Co moments are oriented along the 800 mT magnetic field, and by rotating the FM magnetization in-plane the AFM moments also rotate due to the exchange spring effect. For magnetic tunnel junctions, magneto-transport is governed by magnetic layers directly adjacent to the tunnel barriers. In previous experiments on tunnel junctions with AFM metal layers in contact with the tunnel barrier, e.g. NiFe/IrMn/MgO/Pt, the TAMR results from the AFM layers alone, while the remote FM layer only serves as a means to rotate the magnetic AFM moments in an external magnetic field. In our case, however, the (spin-polarized) electrons participating in the tunnelling process should mainly originate from hybrid interfacial Co/CoO states, and the TAMR results from the interplay of both FM and AFM moments.

In summary, we have investigated TAMR due to spin polarized electron tunnelling through exchange-spring-coupled tunnel junctions. The TAMR signal is governed by the rotation of AFM moments of the insulating CoO barrier rather than the FM moments of the Co electrode alone. Coupling between the AFM and FM moments at the Co/CoO interface, which is evident from the exchange bias effect, leads to an exchange spring effect that results in the rotation of both FM and AFM moments under application of an external magnetic field. The maximum TAMR value observed was 2.5%. A strong bias dependence of the TAMR is observed at 5 K in this device, in particular for the polarity for which electrons tunnel from Co to Al. The largest TAMR (at different angles) was found at non-zero bias currents. We propose that a resonant tunnelling mechanism involving Co/CoO interfacial electronic states plays an important role. Angle-dependent TAMR measurements show that the two-fold symmetry of the TAMR ratio is broken due to the AFM character of the CoO layer. Our results show that it is feasible to control spin-polarized transport via insulating AFM tunnel barriers, and may help pave the way towards the development of spintronic devices based on AFM insulators.

## Methods

### Device fabrication

More details about the fabrication of similar spintronic devices have been published elsewhere^1^,^2^. Briefly, fabrication was carried out by e-beam evaporation through shadow masks inside an ultra-high vacuum (UHV) system equipped with a load-lock chamber where plasma oxidation can be conducted. Using a stainless steel shadow mask, a patterned magnetically soft 8 nm thick Co thin film was grown epitaxially on a single-crystalline sapphire (0001) substrate. The Co layer experienced a partial oxidation process, using an oxygen plasma with an O_2_ pressure of 100 mTorr, at room temperature for 15 mins. In Fig. 1(c), the red and blue curves are the corresponding X-ray reflectivity measurement and the simulated plot (PANalytical, X’Pert Reflectivity) respectively for such a bi-layer, from which the so-obtained CoO thickness was determined. The Néel temperature of the so-fabricated films is ~240 K^21^. A 3.3 nm AlO_x_ tunnel barrier was obtained by deposition of a 2.5 nm Al film followed by 30 minutes plasma oxidation. The finished junction configuration is a vertically layered stack consisting of sapphire(substrate)/Co(4.5 nm)/CoO(4 nm)/AlO_x_(3.3 nm)/Al(35 nm) and the resultant junction area is 250 μm × 300 μm. The schematic drawings of the device can be viewed from Fig. 1(a,b), with arrows indicating the in-plane crystallographic orientations of the fcc-Co film.

### AMR and TAMR measurements

Magneto-transport measurements were performed using a liquid helium flow cryostat equipped with an (maximum 1 Tesla) electromagnet. A four-wire measuring method was adopted for all the following measurements. In order to achieve exchange bias of the Co/CoO heterostructure in a predetermined direction, a 750 mT in-plane magnetic field was applied along the easy axis 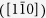 of the Co thin film while cooling down from room temperature to 5 K. A current source and a nanovolt meter were used to input and extract electronic signals. TAMR measurements were performed in two modes. The first was based on injecting a constant *dc* bias current through the junction while recording the voltage upon sweeping the magnetic field along the magnetic easy axis 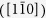 of the Co layer at 5 K. In the second mode, a 800 mT magnetic field was rotated in-plane along different crystallographic axes of the Co thin film. At every angle *θ*, the corresponding *V*–*I* characteristic was recorded and the TAMR ratios were calculated by:


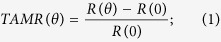


where, *R* represents the resistance for the magnetization applied along a certain in-plane angle *θ*, where *θ* = 0 corresponds to the easy axis 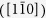.

## Additional Information

**How to cite this article**: Wang, K. *et al.* Tunnelling anisotropic magnetoresistance due to antiferromagnetic CoO tunnel barriers. *Sci. Rep.* **5**, 15498; doi: 10.1038/srep15498 (2015).
